# Neurological and Psychological Effects of Coronavirus (COVID-19): An Overview of the Current Era Pandemic

**DOI:** 10.7759/cureus.8460

**Published:** 2020-06-05

**Authors:** Jawaria Rahman, Abilash Muralidharan, Sohail J Quazi, Hajra Saleem, Safeera Khan

**Affiliations:** 1 Internal Medicine, California Institute of Behavioral Neurosciences and Psychology, Fairfield, USA; 2 Internal Medicine, Kiruba Hospital, Coimbatore, IND; 3 Plastic Surgery, California Institute of Behavioral Neurosciences and Psychology, Fairfield, USA; 4 Plastic and Reconstructive Surgery, Hamad Medical Corporation, Doha, QAT; 5 Family Medicine, California Institute of Behavioral Neurosciences and Psychology, Fairfield, USA

**Keywords:** neurological effects of coronavirus, psychological impacts of coronavirus, coronavirus, covid-19

## Abstract

Coronavirus disease 2019 (COVID 19) is a catastrophic illness that has significantly altered the world’s panoramic view of medicine. As the number of cases around the globe rise, the COVID-19 research writing has been immediately enhanced by professionals internationally. In this review, we focus on the neurological and psychological effects of COVID-19, which can determine both the severity of coronavirus and its related pandemic respectively. While it is critical to distinguish the neurological manifestations from the psychological effects, the latter is becoming more pervasive due to the fast-expanding outbreak.

We conducted a systematic review and included observational retrospective, case-series studies, and surveys to establish the largest pool of valuable research. Articles on these approaches were conducted in PubMed, MEDLINE, and Google scholar. Some gray material was also selected because of the recent nature of the disease.

Data collected from the studies have proposed that COVID-19 is not unusual in demonstrating the neurological symptoms, as it proved in the past by its sister coronaviruses such as severe acute respiratory syndrome coronavirus-1 (SARS-COV-1) and Middle Eastern respiratory syndrome coronavirus (MERS-COV). Studies have presented that some patients with COVID-19 also showed neurological signs, such as headache, nausea, vomiting, dizziness, loss of taste and smell, and impaired consciousness. However, it necessary to clarify that the invasion of severe acute respiratory syndrome coronavirus-2 (SARS-COV-2) directly or indirectly affects the central nervous system (CNS).

Contrarily, the COVID-19 pandemic has affected every single element of life. It has not only changed the individual’s health directly but also has significant psychological, economic, and sociological effects. These issues indicate the disease's extraordinary threat, and we must realize that another pandemic will shortly follow it: that of mental and behavioral illness. Thus, the long-lasting psychological implications of this outbreak deserve further investigation side by side.

## Introduction and background

As coronavirus disease 2019 (COVID-19), and the fears that accompany it, continues to spread around the world, the overall mortality appears to mirror that of pandemic flu [1]. The first instances of an acute respiratory illness, now known as novel coronavirus-infected pneumonia (NCIP), arose in Wuhan, Hubei Province, China [2-3]. COVID-19 has been shown to be a separate group from the previous coronaviruses of the last two decades associated with human SARS and MERS through genomic sequencing and evolutionary differentiation analysis [2]. The percentage of people estimated to have symptoms of this disease when combined with likely infection rates could be as much as 50-80% of the worldwide population [4]. The current death rate of the disease is approximately 3-4% [5].

This novel strain of coronavirus is assumed to have possibly originated in bats before being passed to humans, potentially through another animal, such as a pangolin (scaly anteater). People most likely came into contact with these animals at a so-called “wet market,” where several wild animal species are close to each other for selling purposes. Experts believe that similar new viruses will continue to arise from such places as long as they remain in business [6].

Officials from 192 countries and regions have registered more than 3,620,522 COVID-19 cases and 250,789 deaths since December 2019, when China reported the first cases to the World Health Organization [7-8]. Symptoms including fever, cough, shortness of breath, arthralgias, and fatigue, may appear within 2-14 days after initial contact. Shortness of breath, persistent pain, or feelings of pressure in the chest, as well as novel confusion and inability to arouse are emergency warning signs for COVID-19. Patients with these symptoms should seek immediate medical attention. As of March 2020, no vaccine has been identified yet to prevent COVID-19 [2, 9-10]. One of the more reliable ways to prevent the spread of the virus is to avoid being exposed to it. The infection usually spread mainly through close person-to-person contact. This can also spread through respiratory droplets when a person who is infected coughs or sneezes. The easiest way to prevent disease is by washing your hands or using an alcohol-based sanitizer. Perhaps the population most likely to be affected by the virus are older men with comorbidities [10-11].

According to Kenneth L. Tyler, MD, an author from the American Academy of Neurology, more than one-third of coronavirus patients had some type of neurological manifestations after he observed records from three COVID-19-designated hospitals in Wuhan, China. He mentioned that the symptoms, including acute cerebrovascular disease, evidence of skeletal muscle damage, and altered states of consciousness point towards neurological involvement [12]. He further argued, ʽʽIt makes sense that COVID-19 would have neurologic symptoms because other coronaviruses such as MERS that affect humans can invade the central nervous system”. The neurological symptoms are far more common in severe disease. However, we still don't know whether the neurologic complications are reflective of direct viral injury or due to the secondary or systemic effects of infection [12]. Not much is known about COVID-19’s psychological and neurological consequences; much further work will be needed to get a better understanding.

On the other hand, the circumstances surrounding the ongoing pandemic of coronavirus have led to stressful situations for people and communities. Authorities and companies are heeding warnings regarding social distancing and self-isolation, in order to “flatten the curve” and prevent the spread of the virus. While these measures are necessary to protect the country’s population, they also come with their own attendant issues, such as loneliness, anxiety, and depression. The fear and anxiety regarding the disease have also led to a social stigma regarding certain people, places, and things. For instance, in the early days of the virus, some xenophobic behaviors could be seen as demonstrated by individuals in the form of posts online, comments on videos, and even in-person threats towards people of East Asian ancestry [1]. The focus of this study is to realize the neurological vs. psychological effects of COVID-19.

## Review

Method

We conducted a systematic review and used PubMed, Google Scholar, and MEDLINE for collecting most of the data. Additional data were obtained from the Centers for Disease Control, World Health Organization, and CNN. We carried out our research by using medical subject headings (MeSH) keywords, including "coronavirus", "neurological effects of coronavirus", and "psychological effects of coronavirus". The research mostly pertained to coronavirus effects on the central nervous system (CNS) and mental health issues that developed in communities after the outbreak of COVID-19. Most of the articles used in this study were peer-reviewed; however, some gray literature from government entities and news organizations were included as well to give a fuller scope of the research. This study has chosen to add a population of every age and gender. The articles referenced were accessed as full-text articles. The assessment of multiple systematic reviews (AMSTAR) checklist was used for quality checks for all articles to exclude as much bias as possible. There may be some bias in this study due to the use of gray literature, but this could not be avoided because of the recent nature of the malady outbreak. Overall, the review was done scientifically and ethically.

Result

In total, 54 articles were reviewed, and after quality assessment, 12 were excluded. For neurological effects, five observational retrospective case-series studies have been selected, in which four of them discussed neurological symptoms. The study sample sizes ranged from six patients to 138 patients with study durations of one month to two years. Four studies found neurological symptoms of headache, nausea, confusion, impaired consciousness, and seizures. Psychological effects of the COVID-19 pandemic have been found in very few studies because of the novelty of the disease. After careful consideration, we included two observational survey-based studies, with sample sizes of 1200 to 2000 participants. The survey’s result revealed a broad spectrum of concerns and behavior regarding the impact of the fear and current pandemic of coronavirus across the population. Overall, the neurological and psychological aspects of the viral attack must, therefore, be taken into account in planning the therapeutic strategies and for recuperation paragons aimed at victims of COVID-19.

Discussion

Coronavirus disease 2019 (COVID-19) is a significant hazard to health worldwide. The international community has never faced such a danger to global health within recent memory. This outbreak was announced as a “public health emergency of international concern” by the World Health Organization (WHO) on January 31, 2020. The spread of the disease has consistently increased around the world, reaching more than 100 countries within the first few months of the epidemic. A total of more than 3,620,522 COVID-19 cases and 250,789 deaths have been reported to date by the WHO worldwide [13].

The cases identified outside of China in the initial stages of the international spread of COVID-19 were mainly travelers who came in contact with the virus in China. The United States of America, Malaysia, Japan, Germany, Australia, Singapore, Vietnam, and the Republic of Korea are among the countries that reported COVID-19 cases related to travel. Each country had its response to the initial period of crises. Some immediately put new regulations in place to directly address the spread. Others ignored the initial warnings. Regrettably, domestic spread began in February 2020 in countries such as Italy, Japan, Iran, and South Korea [14]. As an evolving disease, COVID-19 has particular taxonomy, biological attributes, target cells, and clinical symptoms [13].

The Virion

**Figure 1:**
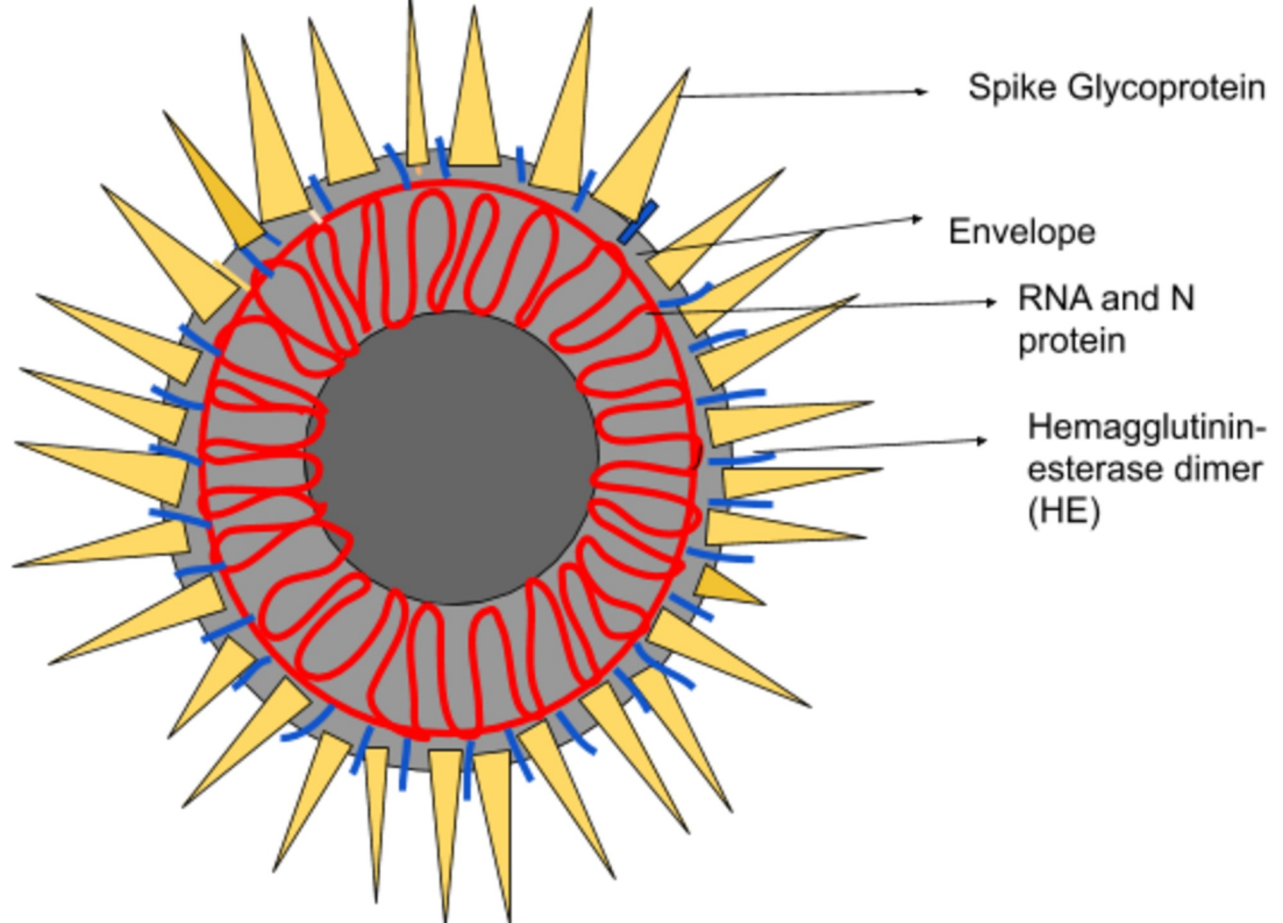
Coronavirus (COVID-19)

The lineage of viruses that COVID-19 belongs to consists of pathogens of many animal species, as well as of humans. This group includes the viruses associated with SARS and MERS. Coronaviruses are a family of enveloped, single-stranded, positive-strand RNA viruses classified within the Nidovirales order [15]. The virion has an enzymatic activity related to hemagglutinin-esterase (HE), a homodimeric class of membrane protein that acts as an invader. Hemagglutinin-esterase assists in the adhesion and dissolution of specific sialic acid receptors that are attached to the host cell surface [16]. The following figure is showing the structure of coronavirus (Figure 1):

Taxonomy

**Figure 2:**
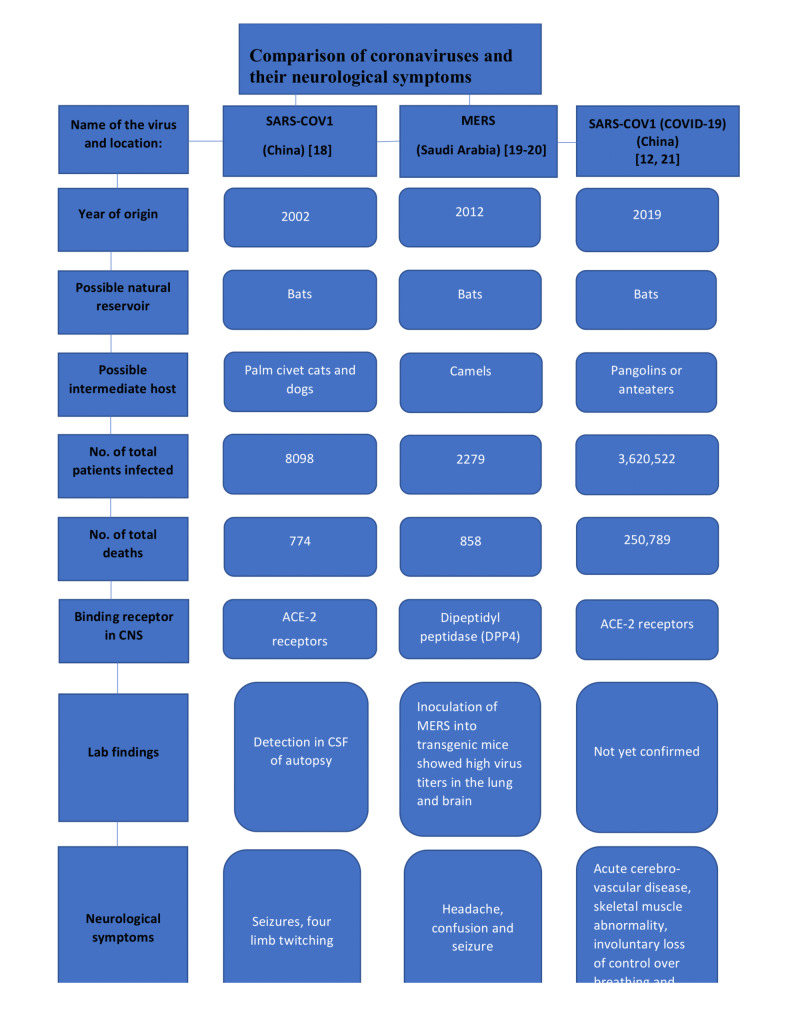
Comparison of different coronaviruses and their neurological symptoms SARS-COV-1: severe acute respiratory syndrome coronavirus-1; MERS: Middle East respiratory syndrome coronavirus; SARS-COV-2: severe acute respiratory syndrome coronavirus-2; ACE-2: angiotensin-converting enzyme-2 receptors; CSF: cerebrospinal fluid

The coronavirus was first classified in the mid-1960s and is dubbed “corona” for the crown-like spikes on their surface. The severe acute respiratory syndrome coronavirus-2 (SARS-CoV-2) belongs to coronaviruses, which also includes severe acute respiratory syndrome coronavirus-1 (SARS-CoV-1), Middle Eastern respiratory syndrome coronavirus (MERS-CoV), human coronavirus- HKU1 (HCoV-HKU1), and human coronavirus-OC43 (HCoV-OC43) [17]. The SARS-CoV-1 and MERS-CoV outbreaks occurred in the years 2002 and 2012, respectively. SARS-Cov-1 and MERS-CoV were the most firmly related coronaviruses in the past saw as naturally neurotropic, as well as have possibly similar natural reservoirs and binding receptors for target cells; along these lines, SARS-CoV-2 can likely have comparative qualities. The comparison of SARS-CoV-1, MERS-CoV, and SARS-CoV-2 has been summarized in Figure 2 below exhibiting their attributes and neurological effects [12,18-21].

Transmission

The dramatic increase in the COVID-19 confirmed cases and deaths is indicative of human-to-human transmission by the virus, although coronaviruses have caused epidemics in which transmission can occur by environmental contamination. The routes of transmission and range of environmental contamination are still unknown, especially for SARS-CoV-2. As per the Centers for Disease Control and Prevention, more information needed concerning the dissemination of the virus; however, as with other human coronaviruses, the spread is thought to occur through person-to-person contact (within six feet) via respiratory droplets [22].

Pathogenesis

The principal target cells for SARS-CoV-2 are epithelial cells of the respiratory and gastrointestinal tract. These cells contain angiotensin-converting enzyme-2 receptors (ACE-2 receptors) which are used by the virus to penetrate the cells. It is unlikely that the invasion of the virus is restricted to only these tissues [23-24]. The scientific data from studies propose ample tissue intrusion and noticeable neurotropism, which can produce yet more intricate clinical scenarios. The question is this: can SARS-CoV-2 invade the CNS and infect neural cells? Assuming the answer is yes, how does the damage to the CNS contribute to its progression, signs, and symptoms, including consequences [23]?

ACE-2 receptors distribution in the brain (Target cells for COVID-19)

The ACE2 receptors present in the glial cells and neurons are potential target cells for COVID-19 in the brain. Past studies have demonstrated the ability of SARS-CoV to cause neuronal demise in mice by invading the brain along with the nose adjacent to the olfactory epithelium [25].

Routes of Invasion in the Central Nervous System (CNS)

The brain usually protects from the free passage of unwanted molecules, pathogens, and cells by the blood-brain and blood-CSF barriers: the first line of defense. The blood-brain barrier is formed of astrocytes, pericytes, extracellular matrix, and cerebral microvascular endothelium. Viral pathogens can use different pathways for CNS invasion, including the hematogenous route-which is the infection of the endothelium and the peripheral nerves or olfactory sensory neurons path [26-27].

*Neurological effects of Coronavirus *(*COVID-19*)

A continuous report regarding the patients demonstrating neurological manifestations of COVID-19 alongside other research findings such as involuntary loss of control over breathing, seizure, headache, and impaired consciousness could clarify the neurotropic potential of the COVID-19 virus [24]. However, there is still a knowledge gap concerning the neurological effects of the illness, and more studies are required to fill in this gap [12].

One of the retrospective case series studies conducted in Wuhan by Mao et al. described some neurological symptoms in patients infected by COVID-19. The data collected between January 16, 2020, and February 19, 2020 and the study included 214 patients. Out of the 214 patients, 78 were severely ill and showed neurological involvement, including acute cerebrovascular diseases, conscious disturbance, and skeletal muscle injury, whereas the other 126 were non-severe patients and displayed no abnormal neurological symptoms [12]. On the other hand, another retrospective case series study conducted by Wang et al. in Wuhan described some CNS abnormalities in both severe and non-severe patients. The study involved 138 patients, and 13 patients reported experiencing dizziness; of these 13 patients, eight were in the intensive care unit (ICU), and five were non-ICU patients. Another nine patients reported headaches; of these, three were ICU patients, and six were non-ICU patients [2]. Analyzing the above two studies showed that contrary to initial research, not only severely ill patients experience neurological symptoms but also patients with less severe manifestations. In the view of the ebb and flow accessible, a better level of evidence should be gathered to expel a specific measure of inclination from the investigation.

In the past, previous studies have established a connection between coronavirus infection and neurological symptoms. In 2005 Gu et al. conducted a case-control study to see the SARS-CoV in multi-organs systems via autopsy. Eight of the 18 suspected SARS victims were confirmed to have SARS through the use of real time-polymerase chain reaction (RT-PCR), electron microscopy, and immunohistochemistry. The autopsy findings have shown clear evidence of the presence of the virus in the cerebrospinal fluid. Edema and red degeneration of the neurons were found in six of the eight confirmed cases of SARS, whereas the pathologic changes were absent in the brains of unconfirmed or control cases [25]. The role of the blood-brain barrier in containing and blocking the virus from acquiring access to the neural tissues requires further research in patients with COVID-19 [28]. Moreover, MERS-CoV, another strand of coronavirus, is a deadly virus with a high mortality rate that can cause severe disease, thus requiring intensive care. One retrospective case series study was conducted in Saudi Arabia in 2014, which included 70 lab-confirmed cases of MERS to study its clinical aspects and outcomes of patients. MERS-CoV symptoms were fever, shortness of breath, and cough, while non-respiratory symptoms included headache (9% of patients), seizure (6%), myalgia (14%), and vomiting [20]. In light of these pieces of evidence, likely, SARS-CoV-2 can also have neurological effects on the body. The various qualities, purposes, and findings of studies explained in the preceding passages have been summed up in the (Table-1) [2, 29, 12, 30-31].

**Table 1:** The neurological symptoms of coronaviruses in relevant studies NCIP: novel coronavirus infected pneumonia; ICU: intensive care unit; CK: creatinine kinase; LM: light microscopy; EM: electron microscopy; MERS COV: Middle Eastern respiratory syndrome coronavirus; RT-PCR: real time-polymerase chain reaction; SARS: severe acute respiratory syndrome

NO:	Study Location	Author and the year of publication	Study Design	Sample Size	Purpose of the Study	Conclusion
1	Wuhan [2]	Dawei Wang et al., 2019 Jan 1 to Jan 28, 2019	Retrospective case series	138	To describe the epidemiological and clinical characteristics of NCIP	Out of 138 patients, 13 got dizziness, in which eight were in ICU, and five were non-ICU admission. Nine patients got a headache in which three were admitted to the ICU and six were non-ICU
2	Shenzhen [29]	Jasper Fuk-Woo Chan et al., 2020 Dec 29, 2019, to Jan 4, 2020	Retrospective case series	Six patients from the same family	To see the familial cluster of pneumonia associated with COVID-19 indicating person-to-person transmission	Patients showed fever, cough, generalized weakness, nasal congestion, rhinorrhea, sneezing, sore throat, pleuritic chest pain, and diarrhea, but no one showed any neurological symptoms.
3	Wuhan [12]	Ling Mao et al., 2020 Jan 16, 2019 to Feb 19, 2020	Retrospective case series	214	To demonstrate the neurological manifestation of hospitalized patients	Out of 214 patients, 78 were in severe condition and showed neurological symptoms as compared to 126 non-critical patients. Neurological symptoms include acute cerebrovascular accidents, impaired consciousness and skeletal muscle symptoms (myalgia and increase CK level)
4	China [30]	Jiang Gu et al., 2005 Jun and Aug 2003 to April 2004	Case-Control	18	To examine the SARS-COV virus in multi-organ systems by demonstrating the pathogen with real-time PCR	Eight of 18 suspected SARs victims were confirmed as having SARS by the demonstration of the pathogen with real-time PCR, in situ hybridization, and electron microscopy. SARS genome sequences were identified in the brain of all SARS autopsies with LM, EM, and RT-PCR. Edema and scattered red degeneration of the neurons were present in the brains of six out of eight confirmed cases of SARS. SARS viral sequences and pathologic changes were not present in the brains of unconfirmed cases or controls.
5	Saudi Arabia [31]	Mustafa Saad et al., 2014 Oct 21, 2012 to May 31, 2014	Retrospective case series	70 lab-confirmed cases	To see the clinical aspect outcome of patients with MERS	MERS COV can cause severe infection requiring intensive care and has a high mortality. Neurological symptoms include confusion 18%, headache 9%, seizure 6%, myalgia 14%.

In the long run, SARs-COV-2 would likely leave permanent neurotoxic effects on the brain. For instance, the Spanish flu outbreak of 1918-19 was associated with a spike in the prevalence of post-encephalitic Parkinsonism [32-33]. It is currently unclear whether the SARS-COV-2 infection will lead to mental health or neurodegenerative maladies immediately after the acute respiratory tract infection has passed, or whether it would take years to manifest. Nevertheless, the action is required at this time is to establish the scientific capabilities to check these fundamental biological causes of cerebral disease associated with COVID-19.

Further investigation should be started in the coming months and years to see if these effects are a result of the primary or the secondary effects of the virus. Also, the data of high transparency need to be gathered to avoid the potential bias by increasing the sample size of the patients researched and the duration of time of the study. With such small sample sizes of patients, the best quality findings cannot be established at the present moment.

Psychological effects of Coronavirus (COVID-19)

The rapid escalation of the COVID-19 and the restrictions of healthcare facilities have caused widespread anxiety and panic among governments, institutions, and individuals in every part of the world. This pandemic has generated an equal level of crisis in countries around the world. Countries that have failed to take proactive measures are now epicenters of the outbreak.

As the COVID-19 epidemic continues, it may have a lifelong psychological impact on the population. A psychology professor, Steven Taylor at the University of British Columbia, is among those who presume from a mental health viewpoint, “many people won’t be going back to normal anytime soon.” Taylor claims that the “coronavirus pandemic could leave psychological scars on people all over the world.” According to Taylor, that trauma will be a form of PTSD: post-traumatic stress disorder” [34].

We have seen increased anxiety, xenophobia, panic-buying, the spread of conspiracy theories, and some instances of theft and robbery. Social distancing and quarantine measures including a lockdown of large cities, mandatory face coverings, shutting borders, and confining people to their homes have been in place since January of 2020, and many will continue for months to come. Unfortunately, by the time these measures were placed, much of the human-to-human transmission had occurred, and the damage had been done. Earlier implementation of these strategies may have been even more effective, and any future outbreaks will benefit from the lessons learned during this time [35]. While these measures are necessary at preventing significant loss of human life, on the other side, they are increasing the psychological strain that many people are currently feeling. Moreover, many businesses have closed down for the foreseeable future, and joblessness rampant, countless people are experiencing acute anxiety, depression, and even suicidal tendencies.

**Figure 3:**
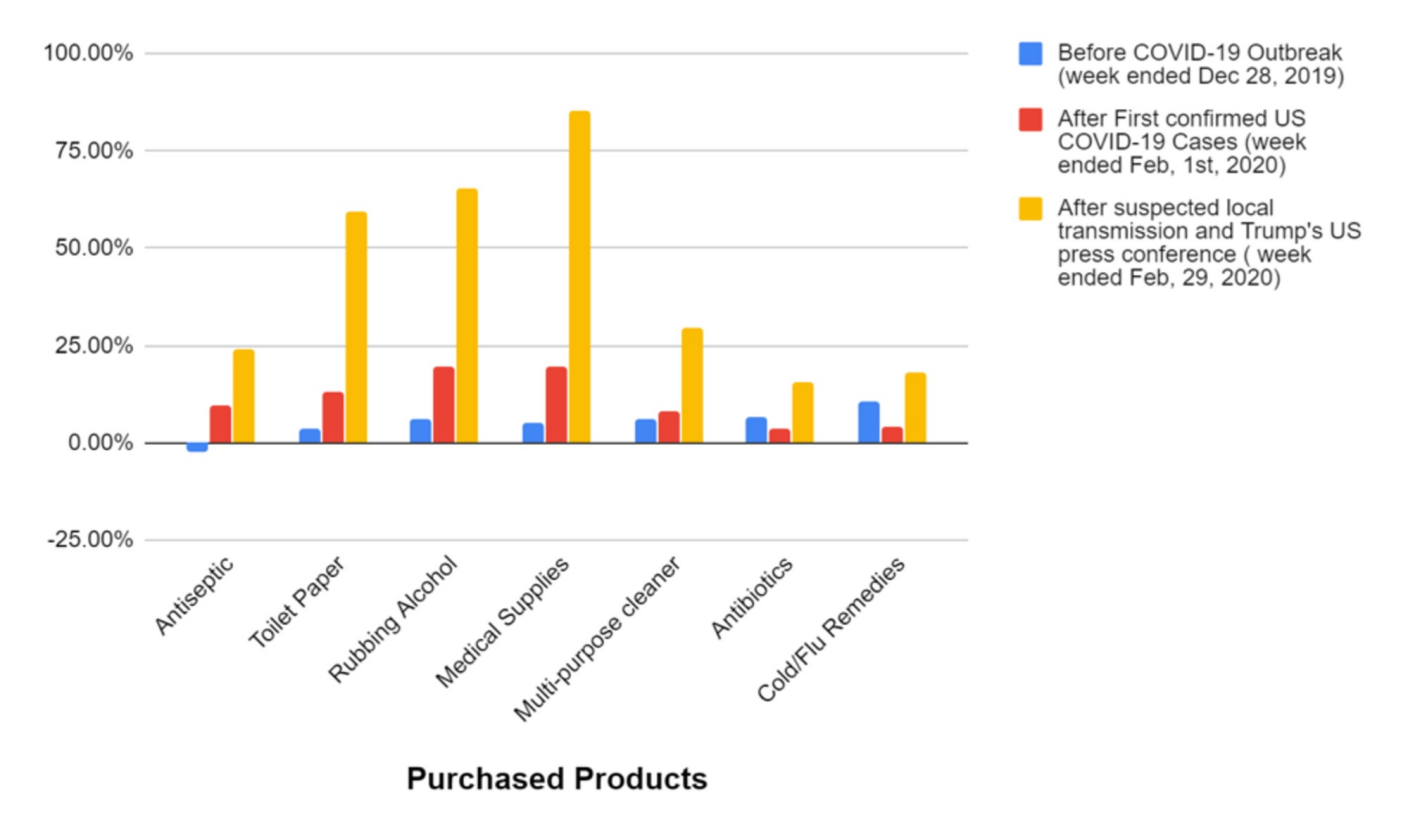
Panic buying after COVID-19 confirmed as pandemic

This pandemic has touched every aspect of modern life. It has not only significant effects on the health of those directly affected by the illness, but also substantial psychological, sociological, and economic effects worldwide [35]. Many people now find themselves isolated from their friends, family, and coworkers while trying to adapt to the new normal of living under social isolation. People have lost significant income from the closure of businesses and the loss of hourly wage jobs. People are reacting to stress in many ways, including panic-buying and stockpiling items for themselves. Stores are routinely running out of milk, eggs, toilet paper, hand sanitizer, medical supplies, antiseptics, and cold and flu remedies. According to the US sales growth, the consumer report by Nielsen compares the panic-buying before COVID-19 outbreak to after the first confirmed US COVID-19 cases in Feb v/s after suspected local transmission and president US press conference which is best elaborated in the graph (Figure 3) [36]. 

This direct lack of empathy shows how this crisis is affecting not only the population socially but psychologically. Others are adapting by becoming more isolated in their thoughts and behaviors than they were before. People are buying guns and other weapons and focusing on the idea of a future war that could come. The population affected by massive disasters are not only left to deal with the immediate physical aftermath and destruction that follow but also forced to reckon with several mental and behavioral disorders that accompany surviving an event of this magnitude. Increases in post-traumatic stress disorder (PTSD), depression, substance abuse, child abuse, and domestic violence have commonly observed [37]. Examples of this were noted in the past, where New Yorkers were affected by the 9/11 attacks [38]. Ten percent of the adult population in New York showed signs of major depressive disorder in the month following the attack. At the same time, one in four of New Yorkers reported an increase in alcohol use. Epidemics can also have a similar effect on those in the closest proximity to the illness. Increases in stress, psychological distress, and PTSD were also reported in 1918-19 after the SARS epidemic among patients and their caretakers [39].

**Figure 4:**
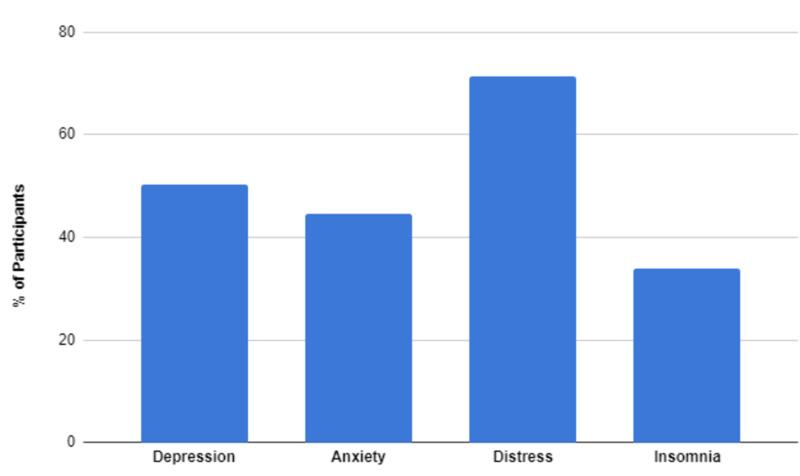
Lai's cross-sectional survey-based study presents percentages of healthcare workers showing mental symptoms during COVID-19 pandemic

One cross-sectional survey-based study, conducted by Jianbo Lai in China, obtained demographic data and mental health assessments from 1257 healthcare workers in 34 hospitals from January 29, 2020, to February 3, 2020. In a total of 1830 individuals, 1257 concluded the survey with an involvement proportion of 68.7%. Of all participants, 493 (39.2%) were physicians, 764 (60.8%) were nurses, and; 760 (60.5%) hospital workers and 522 (41.5%) were first-line health care staff. A substantial number of participants reported symptoms of depression (634 [50.4%]), anxiety (560 [44.6%]), distress (899 [71.5%]) and insomnia (427 [34.0%]) [40]. Lai’s study has been formulated in the following graph (Figure 4).

**Figure 5:**
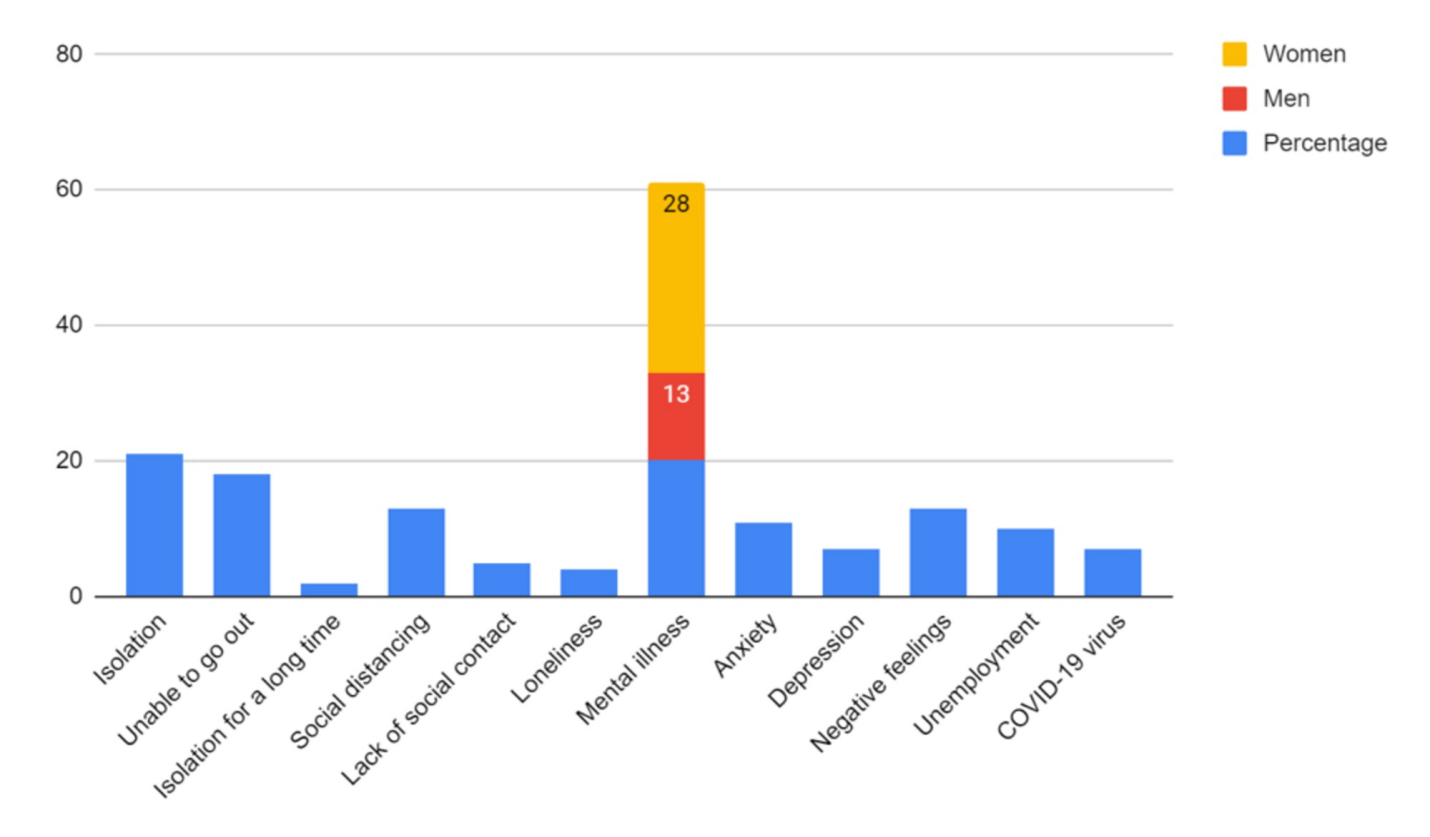
Ipsos MORI survey presenting psychological and mental concerns among the general population during COVID-19 pandemic

Surveys conducted in the UK by the research charity MQ: Transforming Mental Health as well as Ipsos MORI obtained information regarding the mental health concerns and effects of COVID-19. The MQ survey had 2198 participants, who submitted 4350 concerns about the mental health effects of the epidemic and 1987 responses regarding what has maintained their mental health during the outbreak. The data collected in this study may not be representative of the entire population, however, because the majority (80%) of the participants were female, and very few participants were under 18 years old. The Ipsos MORI survey revealed broad patterns of concerns and behavior among the population of the UK. Around 21% are concerned about isolation (of these, 13% are worried about social distancing, with the lack of social contact 5% and loneliness 4%). With a similar number, 20% concerned about mental illnesses (of these 11% worried about anxiety and 7% who have concerns about depression). More than 10% are concerned about negative feelings and the practical aspects of life, such as employment. One in four participants says they are using various forms of entertainment to help their mental well-being during the isolation period. One in five participants stay in touch with family to improve their mental health, while similar numbers are using physical activity [41]. This survey is more representative of the whole population, and trends among the men and women who were surveyed can be noted as well. Women, on the whole, are more likely to be concerned about social distancing, isolation, and mental health illness (28%), while 13% of men were concerned for mental illness [41]. Both surveys and the study contain valuable information; the data collected must be interpreted differently. The Ipsos MORI study is more representative of a country’s entire population and its concerns. In contrast, Lai’s research gives us a glimpse at the direct mental health effects of the pandemic on the healthcare workers closely affected by it. The values of the IPSOS survey have been summarized in the graph (Figure 5).

At the present moment, the world is facing the threat of the most widespread pandemic in recent history, and researchers are speculating that there will be significant increases in the behavioral and psychological disorders that follow disasters. With many people forced to “shelter in place” and schools being closed, there is the possibility of prevalence of child abuse. The concern for such occurrences is so high that the UK has issued psychological first aid guidance from Mental Health UK [42]. The literature, unfortunately, is unclear about comprehensive prevention. Still, it does give people a few steps that can help them to prepare for the increase in mental health conditions that follow this pandemic. An important step is to know that loneliness is inevitable as people physically and socially isolate and that there are ways to help. Using digital technologies, such as video calls and online messaging, can help to create more social spaces for people to interact, even while they cannot be physically close. Many communities and organizations are putting measures in place to allow their members to interact. Online religious services and gym classes, in addition to virtual workspaces, are just some of the steps that have been put in place.

Another crucial step could be training groups and individuals to provide psychological first aid. By teaching people how to provide support to one another through checking in with their family, friends, coworkers, and other community members, individuals can be offered support during the early stages of social isolation. This may create a significant difference in terms of their overall progress. Delivery of care via technology and telemedicine visits by primary providers will also be an essential part of patient care moving forward in the coming months and years [39].

Limitations

The articles included in this review were limited in scope. Most of the studies were from China, where the disease originated, limiting the generalization of our findings to less-affected regions. Also, we cannot ignore the novelty of the disease. Most of the articles of neurological symptoms that were selected for this paper were of short duration, and lack longitudinal follow-up procedures. In regards to psychological research, there has never been a worldwide disaster of this magnitude. Additionally, many people are often reluctant to participate in surveys and studies that relate to mental health issues. Most reviews included were unable to distinguish pre-existing mental health symptoms vs. new symptoms as a response to this virus. A large amount of data will need to be collected, and participants should be made aware of the necessity of the research that is being conducted.

## Conclusions

The neurological symptoms of COVID-19 need investigation as they may have serious implications, but many studies show that psychological symptoms are more prevalent than the neurological. Research is yet unclear whether infection of the brain is a primary or secondary symptom of COVID-19. However, the infection of the central nervous system, mainly the brainstem, could impair the regular functions and the equilibrium of respiration. This hypothesis needs further investigation. If the infection of the brain links to the severity of SARS-COV-2, this will directly affect efforts to treat patients during the current COVID-19 outbreak and will provide the clinicians with a greater understanding of how to manage the most severely ill patients. On the other hand, the world is facing an epidemic of psychological issues that need to be addressed and treated. It is vital to realize the socio-economic effect of the policies used to control the epidemic, which in turn causes a significant adverse impact on mental health. Therefore, future studies about psychological implications will be a valuable investigation. This research review tried to cross-reference both neurological and psychological manifestations of COVID-19, which gives an extensive comparative view of both effects. It provides broad information by analyzing the balance between preventive measures and infection control.
